# Effects of bodyweight support and guidance force on muscle activation during Locomat walking in people with stroke: a cross-sectional study

**DOI:** 10.1186/s12984-020-0641-6

**Published:** 2020-01-13

**Authors:** Jianhua Lin, Guojiong Hu, Jun Ran, Linyu Chen, Xian Zhang, Yanxin Zhang

**Affiliations:** 1 Department of Rehabilitation Therapy, Yangzhi Affiliated Rehabilitation Hospital of Tongji University, No. 2209, Guangxing Road, Songjiang District, Shanghai, 201619 People’s Republic of China; 2 Faculty of Health Sciences, The University of Sydney, Sydney, New South Wales Australia; 3 Department of Exercise Sciences, University of Auckland, Auckland, New Zealand

**Keywords:** Stroke, Locomat, Gait, Electromyography, Rehabilitation

## Abstract

**Background:**

Locomat is a robotic exoskeleton providing guidance force and bodyweight support to facilitate intensive walking training for people with stroke. Although the Locomat has been reported to be effective in improving walking performance, the effects of training parameters on the neuromuscular control remain unclear. This study aimed to compare the muscle activities between Locomat walking and treadmill walking at a normal speed, as well as to investigate the effects of varying bodyweight support and guidance force on muscle activation patterns during Locomat walking in people with stroke.

**Methods:**

A cross-sectional study design was employed. Participants first performed an unrestrained walking on a treadmill and then walked in the Locomat with different levels of bodyweight support (30% or 50%) and guidance force (40% or 70%) at the same speed (1.2 m/s). Surface electromyography (sEMG) of seven muscles of the affected leg was recorded. The sEMG envelope was time-normalised and averaged over gait cycles. Mean sEMG amplitude was then calculated by normalising the sEMG amplitude with respect to the peak amplitude during treadmill walking for statistical analysis. A series of Non-parametric test and post hoc analysis were performed with a significance level of 0.05.

**Results:**

Fourteen participants with stroke were recruited at the Yangzhi Affiliated Rehabilitation Hospital of Tongji University (female *n* = 1; mean age 46.1 ± 11.1 years). Only the mean sEMG amplitude of vastus medialis oblique during Locomat walking (50% bodyweight support and 70% guidance force) was significantly lower than that during treadmill walking. Reducing both bodyweight and guidance increased muscle activity of gluteus medius and tibialis anterior. Activity of vastus medialis oblique muscle increased as bodyweight support reduced, while that of rectus femoris increased as guidance force decreased.

**Conclusions:**

The effects of Locomat on reducing muscle activity in people with stroke were minimized when walking at a normal speed. Reducing bodyweight support and guidance force increased the activity of specific muscles during Locomat walking. Effects of bodyweight support, guidance force and speed should be taken into account when developing individualized Locomat training protocols for clients with stroke.

## Introduction

Gait disturbance is one of the major consequences associated with stroke. Due to the impaired supraspinal control, the gait pattern post stroke is characterized as muscle weakness, spasticity, abnormal muscular amplitude and asymmetrical temporal ordering of muscle activity [1, 2]. Impaired walking ability not only reduces the functional independency of stroke survivors, but also increases a series of risks, like fall [3–5]. The restoration of functional walking ability requires intensive training with a symmetrical gait pattern [6–8].

Various robot-assisted gait trainers, like Locomat, G-EO system Evolution and Gait Trainer, have been designed and implemented in gait rehabilitation for stroke patients [9–15]. These gait trainers enable a repetitive walking training with predefined normal gait pattern and largely reduce the physical demand of therapists [16]. Those robot-assisted gait trainers, like Locomat (Hocoma, Switzerland), can provide a range of adjustable functions, including bodyweight support (BWS), guidance force (GF) and walking speed, allowing clinicians to develop an individualised training protocol that best fits patient’s ability level [17, 18]. Locomat training, however, has been found to reduce muscle activities in both healthy individuals and people with stroke when compared to overground walking [19, 20]. For example, Coenen and colleagues [20] found that the application of BWS and GF significantly reduced activities of several muscles of affected leg in people with stroke. This feature of Locomat training is considered as a negative aspect of its clinical implication because voluntary contraction of muscles plays a key role in motor relearning [21]. In addition, the exoskeletons of Locomat limit the movement in sagittal plane and reduce the degree of freedom of pelvis which may lead to abnormal interaction between the leg and exoskeleton as well as abnormal muscle activity pattern [10, 22].

There is sufficient evidence showing that the Locomat training provided better improvement in terms of independent walking ability, walking speed, balance and disability than conventional physiotherapy to people with stroke [23–28]. There is also evidence that Locomat training significantly improved the duration of single stance phase, step length ratio on the paretic leg when walking on the ground [29, 30]. However, there are also studies showing that the Locomat was not superior to conventional therapy in people with stroke [9, 30, 31]. Despite the heterogeneous features of participants, the difference in training parameters of Locomat may also contribute to the controversial results. In healthy participants, there is ample evidence that BWS or GF can affect the activation of specific muscles [10, 19, 20, 32, 33]. There are also studies reporting significant interactions between BWS, GF and walking speed on voluntary control indicating that the mechanisms of those parameters are complex [32]. In a recent study, however, researchers reported that varying BWS and GF was not associated with changes of muscle activity in people with stroke, whereas increasing walk speed led to greater muscle activity [34]. Since the walking speeds used in previous studies were relatively low (0.56 m/s and 0.61 m/s respectively) [19, 20] and the increase of speed was associated with greater muscle activity [32, 35], it is of interest to investigate whether a higher walking speed would modulate the difference in muscle activity between Locomat walking and treadmill walking.

To further investigate the effects of BWS and GF on active muscle activity, this study aimed to compare the muscle activity level of affected leg between Locomat and treadmill walking at a normal speed in people with stroke. This study also investigated the effects of varying BWS and GF on muscle activity patterns during Locomat walking. Therefore, we hypothesized that when walking at a normal speed, people with stroke exhibit lower muscle activity in the affected leg during Locomat walking than during unrestrained treadmill walking. We also hypothesized that reducing BWS and GF will increase muscle activity level of the affected leg in people with stroke.

## Methods

This was a cross-sectional study which compared the muscle activity of affected leg among different walking conditions in people with stroke. Participants were recruited from inpatients that were receiving rehabilitation at Shanghai Yangzhi Affiliated Rehabilitation Hospital of Tongji University between 13 July 2017 and 29 June 2018. A senior physiotherapist (GJH) was responsible for eligibility screening. Participants were eligible for this study if they were post stroke, aged 18 years or above, had abnormal gait but could walk independently at 1.2 m/s without assistance, and had no Locomat training experience. The abnormal gait in present study refers to the gait that is characterized by compensative movement pattern due to reduced selective motor control (like leg circumduction, pelvic hiking, drop foot during swing phase), asymmetrical spatial and temporal performance (like reduced stride length, shortened single stance time of affected leg and increased step width). Participants were excluded if they had trauma or surgical history in lower limb, severe osteoporosis, cognitive impairment and pathological complications, or had pain during walking. Written informed consents were obtained from all participants before testing.

### Experimental protocols

Prior to trials, detailed instruction and explanation of the experiment was provided to each participant. Participants were first asked to complete a treadmill walking trial without exoskeleton. Subsequently, they performed four walking trials with Locomat under different combinations of BWS (30% or 50% of participant’s body weight) and GF (40% or 70%) in a randomized order (see Table 1). The walking speed for all trials was set at 1.2 m/s. For each trial, participants walked for 3 min. The first 2 min walking served as warm up allowing participants to get used to the walking condition while the last minute walking was used for analysis. Participants were allowed to take an at least 3-min break between two trials. Elastic foot lifter was applied to all participants during walking in the Locomat. The movement of affected leg was recorded for all walking conditions (frame rate = 30 fps).

**Table 1:** Walking conditions of Locomat trials

Condition	Bodyweight support	Guidance force
1	50%	70%
2	50%	40%
3	30%	70%
4	30%	40%

### Surface electromyography recording and analysis

**Fig. 1:**
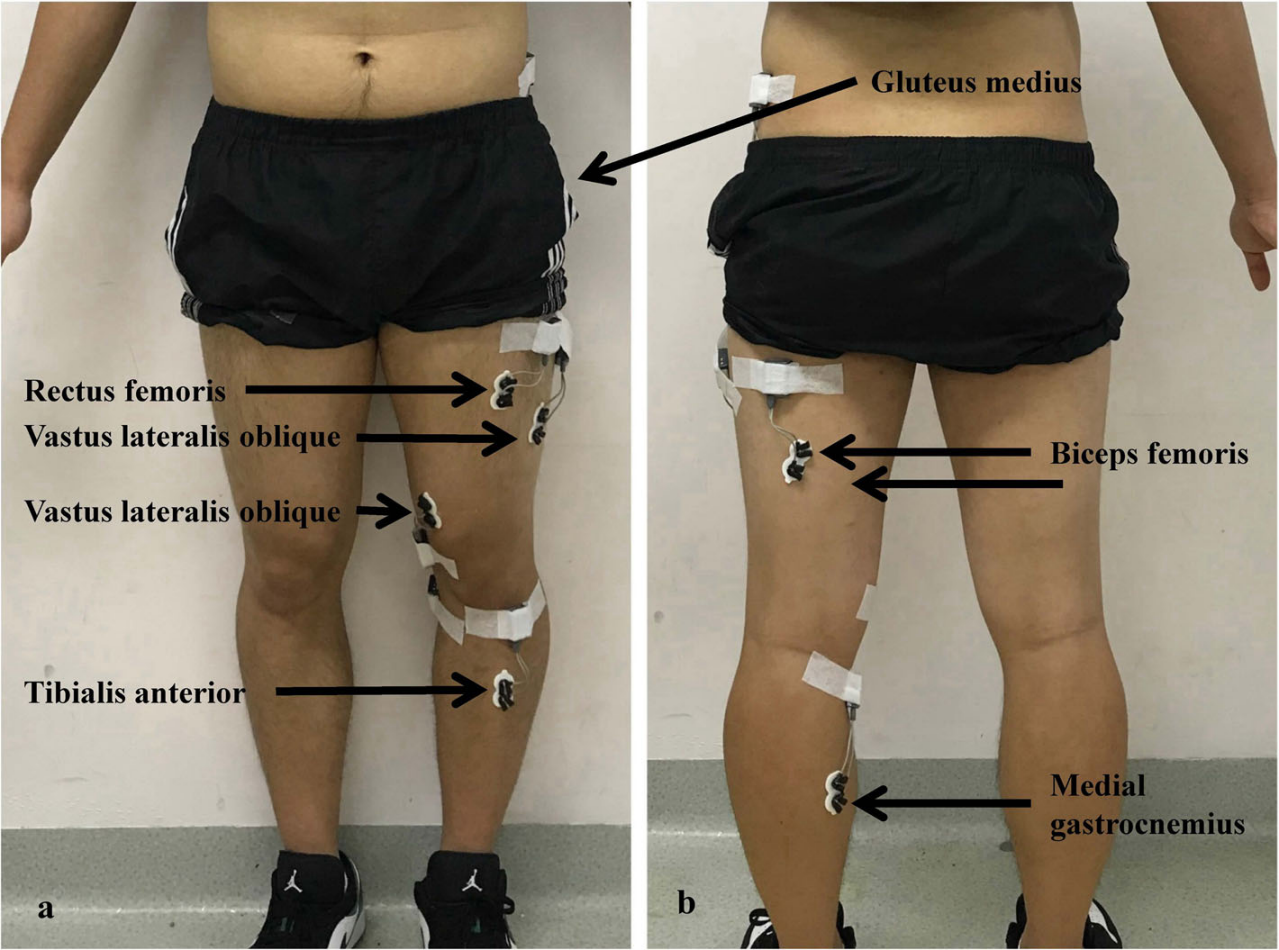
Placement of electrodes. **a**: the front view; **b**: the back view

Muscle activities of affected leg during walking were recorded by using surface electromyography (sEMG) (Noraxon U.S.A., Inc.) from gluteus medius (GM), vastus medialis oblique (VMO), vastus lateralis oblique (VLO), rectus femoris (RF), biceps femoris (BF), medial gastrocnemius (MG) and tibialis anterior (TA) (see Fig. 1). Prior to the placement of electrodes, the body hair in the electrode sites was shaved and then the skin was abraded and cleaned [36]. The disposable Ag/AgCl electrodes (272S, Noraxon USA, Inc., Scottsdale, AZ, USA) with a 10 mm diameter and a 20 mm inter electrode distance were placed parallel to the muscle fibre according to the SENIAM recommendations [37]. The sEMG sampling frequency was set at 1500 Hz. The sEMG signal was synchronized with the video of walking trials.

The raw sEMG signal was first processed by a 20 Hz second-order high-pass Butterworth filter and then rectified. A 4 Hz fourth-order low-pass Butterworth filter was applied afterwards. Then the root-mean-square envelope of the sEMG signal was calculated using a moving window (100 ms). The amplitude of sEMG envelope under each walking condition was normalised with respect to the peak amplitude during unrestrained treadmill walking [34].

**Fig. 2:**
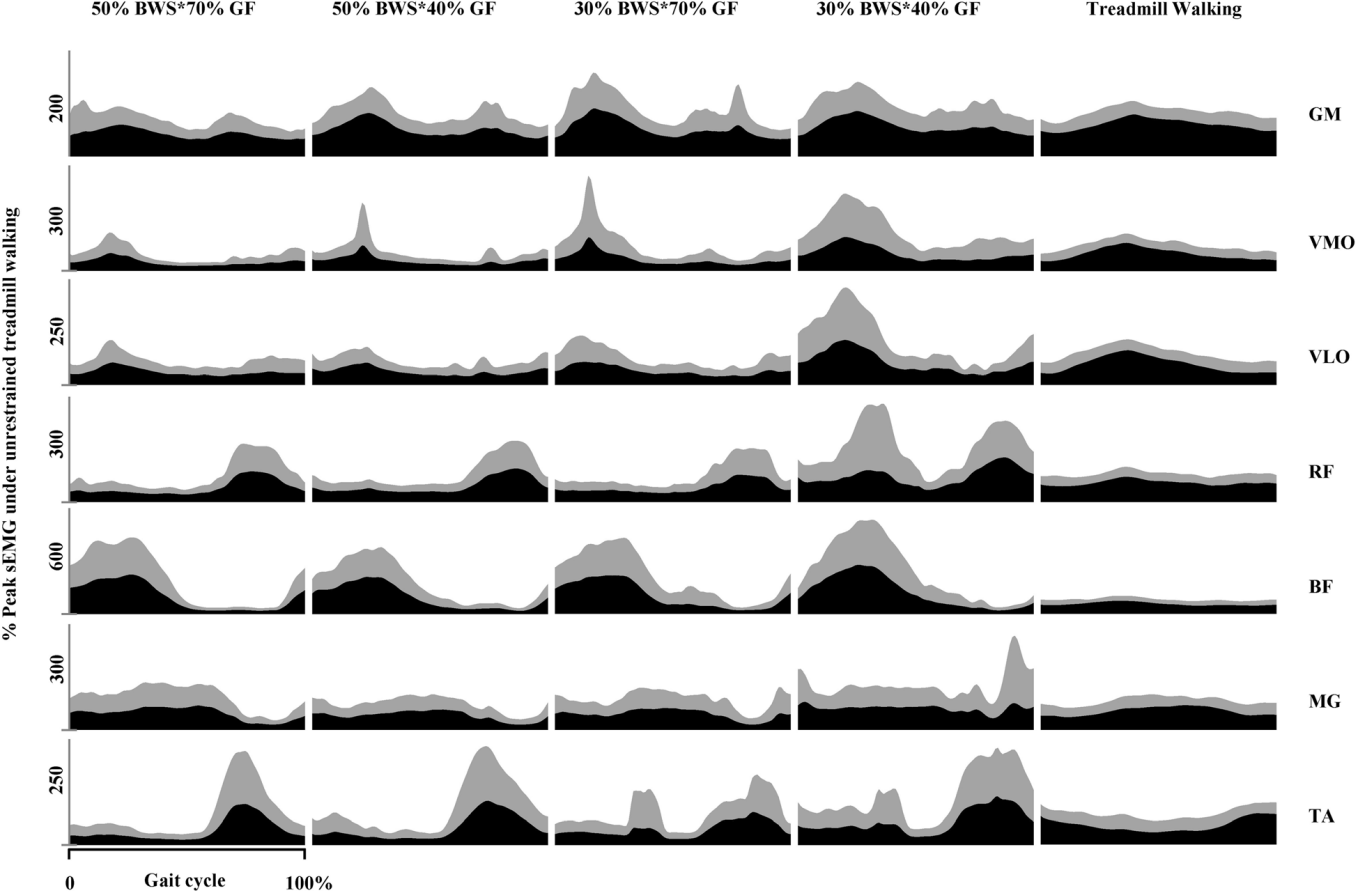
Averaged sEMG profiles during a gait cycle. The black and grey areas represent mean and standard deviation of sEMG. sEMG: surface electromyography; BWS: body weight support; GF: guidance force; GM: gluteus medius; VMO: vastus medialis oblique; VLO: vastus lateralis oblique; RF: rectus femoris; BF: biceps femoris; MG: medial gastrocnemius; TA: tibialis anterior

The gait cycle was defined as the duration between two consecutive heel strikes [19]. The heel strikes were detected by manual inspection of the video of walking trials. The sEMG data of each gait cycle was time normalized into 100 data points. The mean sEMG amplitude of the gait cycle was calculated as the mean value of the 100 data points and averaged over the gait cycles for each muscle and each participant. The averaged mean sEMG amplitude was used for statistical analysis. A figure of sEMG profiles over a gait cycle was created to display the averaged muscle activity pattern of each muscle under each walking condition (see Fig. 2).

### Statistical analysis

Mean and standard deviation were calculated for age, course post stroke and mean sEMG amplitude. The mean sEMG amplitudes under all five conditions were compared by using Friedman test as the sphericity assumption for repeated measures ANOVA was violated for all muscles. Post hoc analysis was performed by using Wilcoxon signed-rank test with Bonferroni correction for multiple comparisons. The statistical analysis was performed with IBM SPSS Statistics 22. The significance level for Friedman test was set at 0.05 and the significance level for post hoc test was set at 0.005.

## Results

**Fig. 3:**
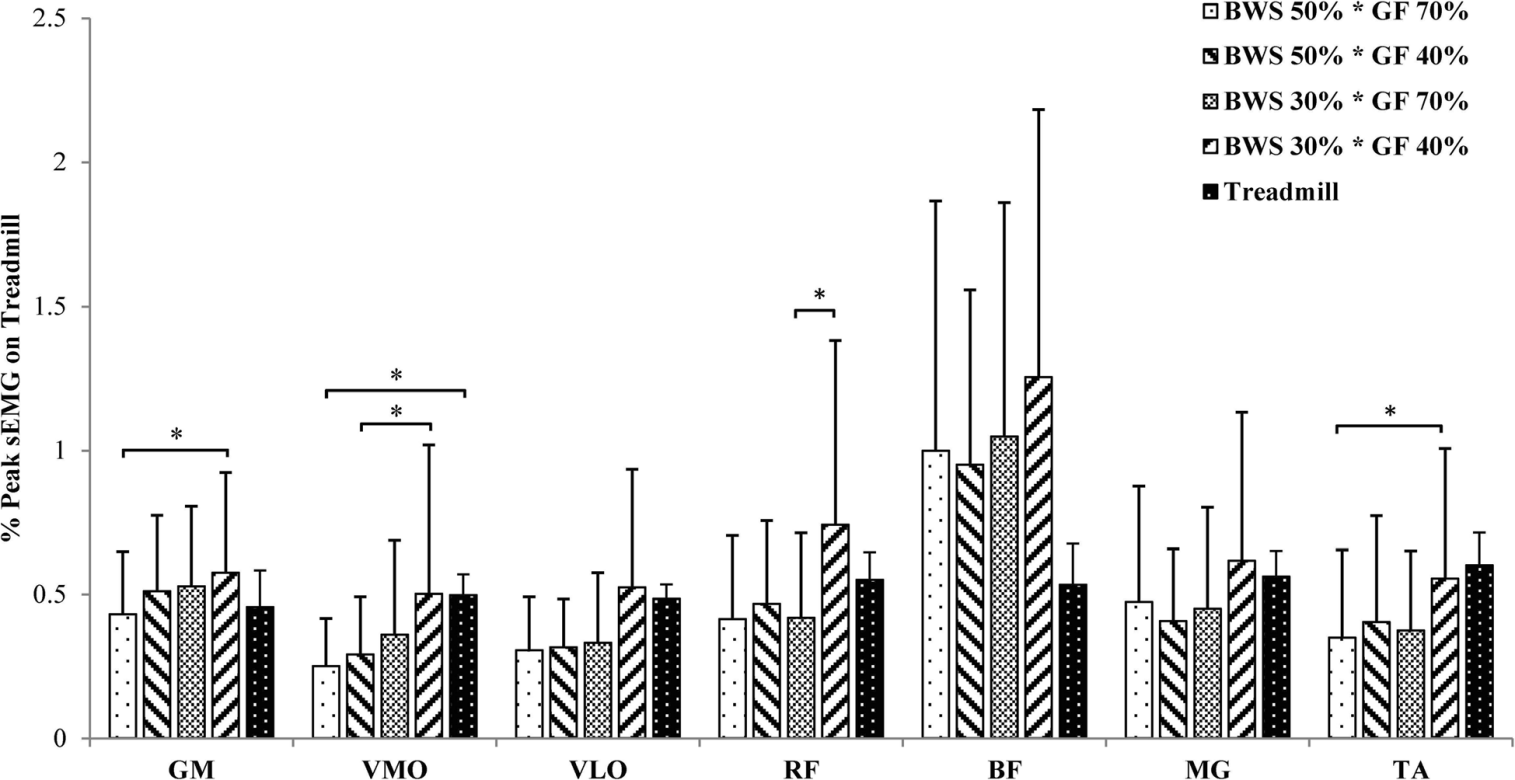
Mean and standard deviation of normalised muscle activity amplitude. sEMG: surface electromyography; BWS: body weight support; GF: guidance force; GM: gluteus medius; VMO: vastus medialis oblique; VLO: vastus lateralis oblique; RF: rectus femoris; BF: biceps femoris; MG: medial gastrocnemius; TA: tibialis anterior

Fourteen participants were recruited for this study. The demographic data are shown in Table 2. The averaged sEMG pattern over a gait cycle is presented in Fig. 2. The mean sEMG amplitudes of each muscle under all walking conditions and their comparisons are shown in Fig. 3. Although significant within-subject changes were revealed by Friedman test for all muscles, only a few significant changes were showed by post hoc analysis.

**Table 2:** Demographic characteristic of participants

Participant number	Gender	Age (years)	Affected side	Stroke type	Time since stroke (months)
1	Male	36	Left	Infarction	1.5
2	Male	32	Right	Haemorrhage	9.3
3	Male	59	Left	Infarction	2.1
4	Male	36	Right	Infarction	1.2
5	Male	52	Right	Infarction	12.1
6	Female	69	Left	Infarction	1.6
7	Male	33	Left	Infarction	2.5
8	Male	38	Right	Haemorrhage	6.7
9	Male	35	Right	Infarction	1.9
10	Male	57	Left	Infarction	10.2
11	Male	45	Left	Haemorrhage	3.6
12	Male	52	Left	Infarction	31.4
13	Male	52	Left	Infarction	5.7
14	Male	52	Right	Haemorrhage	8.9
Mean ± SD		46.29 ± 11.48			7.05 ± 7.93

### Comparison between Locomat walking and unrestrained treadmill walking

Most muscles exhibited similar activation patterns during Locomat walking to that during treadmill walking except BF, RF and TA (see Fig. 2). The activation level of BF during the first half of gait cycle during Locomat walking was remarkably higher than that during treadmill walking. An obvious increase of activation level was also found in RF and TA during the latter half of gait cycle during Locomat walking.

Most muscles demonstrated lower mean sEMG amplitudes during Locomat walking (except under 30% BWS and 40% GF) than unrestrained treadmill walking, except the GM and BF (see Figs. 2 and 3). However, only the difference in mean sEMG amplitude of VMO between Locomat walking (with 50% BWS and 70% GF) and unrestrained treadmill walking was statistically significant (see Fig. 3).

### Effects of varying bodyweight support and guidance force during Locomat walking

In general, the mean sEMG amplitude of muscles tended to increase as the BWS and GF decreased (see Figs. 2 and 3). GM and TA demonstrated significant increases of mean sEMG amplitude when BWS and GF decreased from 50 to 30% and from 70 to 40% respectively. When GF was at 40%, mean sEMG amplitude of VMO increased significantly as BWS decreased from 50 to 30%. When BWS was at 30%, reduction of GF was associated with a significant increase of mean sEMG amplitude in RF. No other significant change was found for the rest muscles.

## Discussion

The present study found that the application of BWS and GF during Locomat walking only reduced the muscle activity in VMO compared to unrestrained treadmill walking in people with stroke when a normal speed was selected. The results of this study also showed that reducing BWS and GF led to increased muscle activities in GM, VMO RF and TA.

### Locomat walking vs. unrestrained treadmill walking

Although Locomat walking generally tended to reduce the mean activity level in most of the muscles, the present study showed that it only significantly reduced the activity level of VMO compared to unrestrained treadmill walking when a normal walking speed was selected. This finding was inconsistent with previous studies where significant reduction of muscle activity was found in GM, VLO, RF, MG and TA in people with stroke [19, 20]. Since the settings of BWS and GF in present study were similar to their studies, the different results may be attributing to the different walking speeds between studies. The walking speed in their studies were 0.56 m/s and 0.61 m/s [19, 20], which were much lower than that in our study (1.2 m/s). Sufficient evidence has shown that higher walking speed was associated with greater muscle activity as to meet the higher demand of energy output [34, 35, 38–41]. The different findings between studies may suggest that the effects of Locomat training on reducing muscle activity in people with stroke may be eliminated by using a normal walking speed.

This study also found that GM and BF showed higher mean sEMG amplitudes during Locomat walking, although the differences were not statistically significant. In theory, offering BWS and GF during Locomat walking could facilitate movement control of legs by reducing muscle load. However, the effects of Locomat walking on muscle activity are inconsistent in the literature. Some studies have shown that Locomat walking was associated with lower muscle activity compared to treadmill walking in both healthy individuals and people with stroke [19, 20], while other studies have found that Locomat walking increased muscle activity in several leg muscles, including BF, quadriceps and gluteus muscles in heathy participants [10, 42]. As suggested in previous studies, the increase of muscle activity may be the results of interaction between the active leg movement and the inertia of the exoskeleton [10, 43]. During Locomat walking, the built-in gait patterns according to which the exoskeletons drive leg movements may restrict the active leg movement in sagittal plane and the pelvic movement which can be confirmed by the interaction force between participants’ leg and exoskeleton [10]. Therefore, greater muscle activity will be exerted when the exoskeletons resist the active leg movement. For example, in present study, the activity of BF during Locomat walking in the first half of gait cycle was relatively higher than that during treadmill walking. This may result from the interaction between the leg and exoskeleton when the knee movement was not compliant with the built-in gait pattern.

### Effects of varying BWS and GF on muscle activity during Locomat walking

The present study has revealed that increasing BWS and GF could reduce muscle activity in specific muscles which are in light with some previous studies on healthy individuals [32, 42, 43]. However, in a recent research on stroke [34], authors reported that BWS and GF had little effect on muscle activity. The different results may not be directly related to the magnitude of change in BWS and GF as the magnitude of change in BWS and GF in our study were 20% (30 and 50% of body weight) and 30% (70 and 40% of guidance force) respectively, whereas that in previous study were 50% (0 and 50% of body weight) and 50% (50 and 100% of guidance force) respectively. But the magnitude of change in muscle activity may be related to the level of GF. van Kammen et al. [34] speculated that more voluntary muscle contraction may be stimulated if the guidance level is lower than 50%. This speculation could be supported by the present study and other studies where one of the GF settings was lower than 50% and significant changes were reported [32, 33, 43]. This evidence indicates that there may be a threshold of GF under which the active muscle activities may increase during Locomat walking. Another potential explanation for the different findings may be that the participants in our study walked at a faster speed (1.2 m/s) than theirs (0.56 m/s) as higher walking speed can lead to greater muscle activity [34, 35, 38–41].

Moreover, the muscles affected by varying BWS and GF in current study were inconsistent with those in previous studies on healthy individuals. For example, in present study, reducing GF led to increased mean sEMG amplitude of RF while similar effects were reported on erector spinae, gluteus medius, biceps femoris, gluteus medius and tibialis anterior in previous studies on healthy individuals [32, 33, 43]. The different results of studies may attribute to the different muscle synergies, as the participants in these previous studies were neurologically intact while that in present study were with stroke.

The findings in this study may be limited by several factors. First, the participant gender is not balanced as only one female participant involved, which may introduce a bias. Second, participants in this study were at different courses post-stroke which made the results not generalizable to specific stroke population. There is compelling evidence showing that the most walking function recovered in the acute stage post-stroke [44–47]. Although the underlying mechanisms remain unclear, the recovery pattern of motor function post stroke suggests that course of post-stroke plays a significant role in recovery of motor function. In other words, the people with acute stroke may respond differently to the same Locomat setting when compared to people with chronic stroke. The future study should address the effects of course post-stroke by using specific stroke population and a longitudinal design. Third, the walking function of the participants was relatively good as they could walk unrestrained on a treadmill at a speed of 1.2 m/s. The results in this study may not apply to the participants with lower walking capacity. Fourth, the walking speed used in present study was relatively high for participant with stroke. The present findings may not reflect the effects of BWS and GF at a lower walking speed as there may be interactions between those parameters [32]. In addition, the temporal characteristics of muscle activation were not investigated in present study which made the effects of BWS and GF on the muscle activity level during each phase of gait cycle unknown. In order to demonstrate temporal characteristics of sEMG profile, a figure of mean sEMG amplitude over a gait cycle was provided to enable visual comparisons between different walking conditions. However, the gait cycle during Locomat guided walking is modulated by the build-in gait pattern and presents different duration of each phase compared to that during unrestrained treadmill walking. As aforementioned, the physical constraints by Locomat may lead to abnormal interaction between the legs and exoskeletons as well as abnormal muscle activity pattern [10, 22]. Those factors may limit the significance of comparing temporal characteristics of muscle activity between Locomat guided walking and treadmill walking. Finally, the changes of muscle activity observed in present study were immediate effects rather than long-term effects. To better examine the effects of Locomat training on muscle activity, future research should measure both the spatial and temporal characteristics of muscle activity with long-term follow-ups.

The findings in present study may suggest that a normal walking speed should be selected for people with stroke during Locomat training. Locomat guided walking has been commonly reported to reduce muscle activity which is viewed as a negative aspect of its clinical implication because voluntary contraction of muscle plays a key role in motor relearning [21]. The findings in our study showed that when a normal walking speed was used for Locomat training, its effect on reducing muscle activity was minimized. The present study also suggested that clinicians could modulate the activities of specific muscles by adjusting BWS and GF in people with stroke. More specifically, if the training target is to reduce undesirable activities in GM, VMO, RF and TA, higher BWS and GF should be selected. On the other hand, if higher voluntary muscle activity is desired, then lower BWS and GF should be employed. However, individuals may respond to the same Locomat training protocol differently. For example, the maximal muscle activity occurred at different combination of guidance force and body weight support in different patients [48]. Given that the interaction between active leg movement and exoskeleton could modulate the neuromuscular control, it would be of interest to clinical practice to investigate the role of the interaction between leg and exoskeleton in modulating muscle activity at each phase during a gait cycle, as well as its association with BWS, GF and speed.

## Conclusions

The present study showed that the effects of Locomat on reducing muscle activity in people with stroke were minimized when walking at a normal speed. This study also revealed that reducing bodyweight support and guidance force increased the activity amplitude of specific muscle groups during Locomat walking. The findings of this study would suggest that effects of bodyweight support, guidance force and speed should be taken into account when developing individualized Locomat training protocols for clients with stroke.

## Data Availability

The datasets used and/or analysed during the current study are available from the corresponding author on reasonable request.

## Abbreviations

BF: Biceps femoris
BWS: Bodyweight support
GF: Guidance force
GM: Gluteus medius
MG: Medial gastrocnemius
RF: Rectus femoris
sEMG: Surface electromyography
TA: Tibialis anterior
VLO: Vastus lateralis oblique
VMO: Vastus medialis oblique
